# The Use of the Lumbosacral Enlargement as an Intrinsic Imaging Biomarker: Feasibility of Grey Matter and White Matter Cross-Sectional Area Measurements Using MRI at 3T

**DOI:** 10.1371/journal.pone.0105544

**Published:** 2014-08-29

**Authors:** Marios C. Yiannakas, Puneet Kakar, Luke R. Hoy, David H. Miller, Claudia A. M. Wheeler-Kingshott

**Affiliations:** NMR Research Unit, Queen Square MS Centre, Department of Neuroinflammation, UCL Institute of Neurology, London, United Kingdom; University Medical Center (UMC) Utrecht, Netherlands

## Abstract

Histopathological studies have demonstrated the involvement of spinal cord grey matter (GM) and white matter (WM) in several diseases and recent research has suggested the use of magnetic resonance imaging (MRI) as a promising tool for *in vivo* assessment of the upper spinal cord. However, many neurological conditions would benefit from quantitative assessment of tissue integrity at different levels and relatively little work has been done, mainly due to technical challenges associated with imaging the lower spinal cord. In this study, the value of the lumbosacral enlargement (LSE) as an intrinsic imaging biomarker was determined by exploring the feasibility of obtaining within it reliable GM and WM cross-sectional area (CSA) measurements by means of a commercially available MRI system at 3 tesla (T). 10 healthy volunteers (mean age 27.5 years, 6 female) gave written informed consent and high resolution images of the LSE were acquired and analysed using an optimised MRI acquisition and analysis protocol. GM and WM mean CSA measurements were obtained from a 15 mm section at the level of the LSE and the reproducibility of the measurements was determined by means of scan-rescan, intra- and inter-observer assessments. Mean (±SD) LSE cross-sectional area (LSE-CSA) was 62.3 (±4.1) mm^2^ and mean (±SD) LSE grey matter cross-sectional area (LSE-GM-CSA) was 19.8 (±3.3) mm^2^. The mean scan-rescan, intra- and inter-observer % coefficient of variation (COV) for measuring the LSE-CSA were 2%, 2% and 2.5%, respectively and for measuring the LSE-GM-CSA were 7.8%, 8% and 8.6%, respectively. This study has shown that the LSE can be used reliably as an intrinsic imaging biomarker. The method presented here can be potentially extended to study the LSE in the diseased state and could provide a solid foundation for subsequent multi-parametric MRI investigations.

## Introduction

Many neurological conditions cause intrinsic pathological changes in the spinal cord (SC) [1]. Some conditions like Friedrich's ataxia (FRDA) and leucodystrophies will only affect the white matter (WM) tracts of the SC [2], [3] while others, such as amyotrophic lateral sclerosis (ALS) and multiple sclerosis (MS), will involve both the grey matter (GM) and WM [4]–[7]. The variety and discriminating behaviour of intrinsic SC pathologies has profound clinical implications; purely GM lesions (sparing surrounding WM) cause clinical dysfunctions that correspond to the level of the pathological changes, while WM tract destruction will lead to motor, sensory and sphincter symptoms below the level of the damage [8].

Previous research has used cross-sectional area (CSA) measurements of the upper cervical cord, obtained by means of magnetic resonance imaging (MRI), to assess the degree of atrophy (i.e. tissue loss, which implies neurodegeneration). This measure has been significantly correlated with measures of locomotor disability in people with MS [9]–[11], while a decline of spinal cord CSA over time of between 11% and 30% has also been observed in cases of chronic spinal cord injury (SCI) using similar methodologies [12]–[14]. While measuring atrophy and cross-sectional dimensions provides an index of overall damage to the cord over time, it does not elucidate between individual rates of tissue (WM and GM) loss, which may have prognostic implications with respect to both disease progression rates and response to treatment. The use of MRI to assess tissue-specific changes within the SC has been limited due to a number of technical factors relating to resolution, signal-to-noise ratio (SNR) and motion artefacts, which make it technically difficult to assess the integrity of the SC in detail [15].

Recent research has shown the potential to segment GM and WM reliably within the healthy cervical spinal cord [16] but evidence from *ex vivo* investigations has shown that loss of somatic and motor neurons may also occur in the lower spinal cord and maybe best studied independently when trying to address clinical questions [17]. The need to develop new imaging methods to gauge tissue-specific changes in the lumbar region is therefore profound, as it would allow important neurological conditions such as the aforementioned to be studied in detail. However, the study of the lumbar SC poses additional challenges which merit investigation in their own right. For example, the cord segments in the lumbar SC do not correspond consistently with the vertebral body levels [18], [19] and these positional variations of the SC must be accounted for. In addition, technical considerations such as the management of patient related and physiological motion, or identifying the correct balance between image resolution, SNR and scan time, are all equally important determinants of a clinically useful imaging protocol.

In this study, a commercially available MR sequence and image analysis software are utilised in a protocol for GM and WM segmentation in the lumbar SC, which takes into account the technical challenges associated with imaging the lumbar SC. One of the innovative strategies employed in this study is to take into account the positional variations of the SC through the use of the lumbosacral enlargement (LSE) as an intrinsic imaging biomarker, owing to its consistent relationship with the T11 - L1 lumbar SC segments [20]. Positional variations in the lower SC have been studied previously in the context of providing important safety information in spinal anaesthesia for preventing intrathecal needles from being directed close to the cord, mainly by reporting the termination level of the conus medullaris [21]–[23]. The conus medullaris extends caudally from the LSE and becomes gradually narrower and thinner. The LSE is a large enough structure that can be studied reliably with clinically available MR systems. This work assesses the reproducibility of the proposed MR imaging and analysis protocol and provides normative data hitherto unreported *in vivo*, serving as a foundation for further multi-parametric MR investigations in the lumbar SC.

## Materials and Methods

### Study participants

Ten healthy adult volunteers were recruited for the study (mean age 27.5 years, 6 female, range 21–35). Written informed consent was obtained from all participants and the work was approved by the National Hospital for Neurology and Neurosurgery and the Institute of Neurology Joint Research and NRES Committee London Bloomsbury (Formally London REC 2 Ethics Committee).

### MR imaging

**Figure 1:**
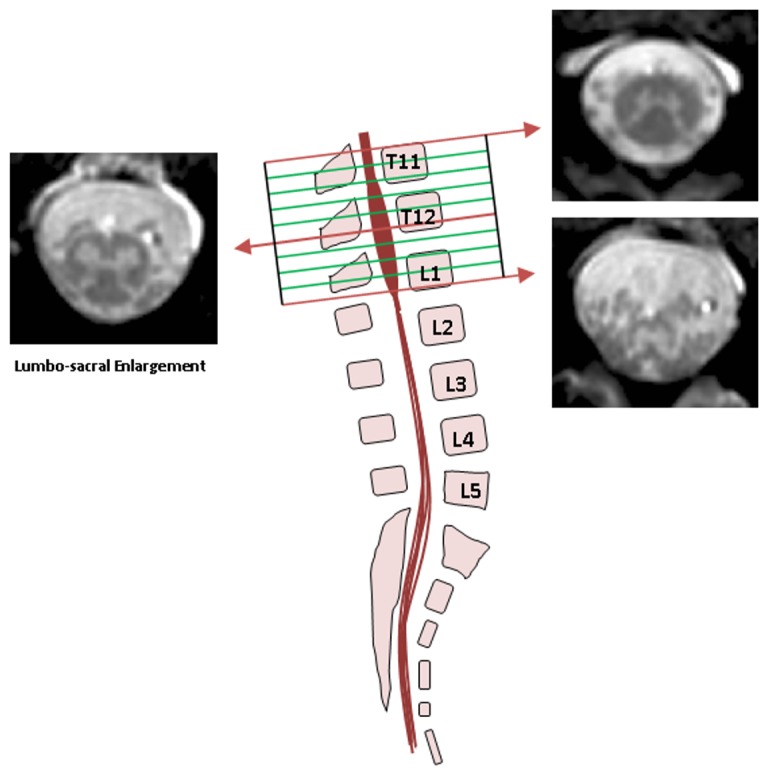
Imaging the lumbosacral enlargement (LSE). The imaging volume was prescribed to cover from the superior margin of T11 to the inferior margin of the L1 vertebral bodies so that to ensure coverage of the LSE in all subjects. Example images are shown from superior, middle and inferior sections of the imaging volume.

A 3T Philips Achieva MRI system was used with dual-transmit technology (Philips Healthcare, Best, Netherlands) using the manufacturer's 16-channel neurovascular (NV) coil and 15-channel SENSE spine coil. Considering previously reported imaging protocols that offered high image contrast in the upper spinal cord [16], [24], the lumbar SC was imaged in the axial plane with the slices perpendicular to the cord using a fat-suppressed 3D slab-selective fast field echo (FFE) sequence and the following acquisition parameters (for acquisition parameter optimisation details refer to Appendix S1): Repetition time (TR) = 23 ms; echo time (TE)  = 4.4 ms, flip angle α = 10°, field of view (FOV)  = 180×180 mm^2^, voxel size  = 0.5×0.5×5 mm^3^, number of averages (NEX)  = 8, 19 axial contiguous slices and scanning time of 19∶27 min. The first slice of the imaging volume was positioned at the superior margin of the T11 vertebral body with the volume extending minimally to the inferior margin of the L1 vertebral body to ensure coverage of the LSE in all subjects (see Figure 1).

Motion artefacts were reduced by using velcro straps to restrain the torso and through the use of foam padding to reduce inadvertent movements of the upper neck. Hip flexion was achieved through the use of a large foam wedge that increased the level of contact between the lower back and the coil surface. Every effort was made to ensure the participants were as comfortable as possible in the scanner.

### Image analysis

**Figure 2:**
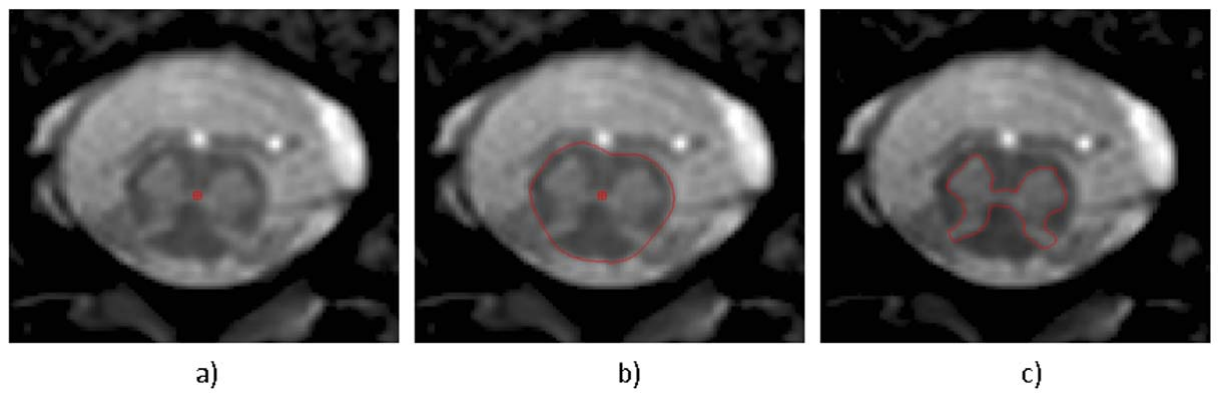
Segmentation of the lumbosacral enlargement (LSE) using the active surface model (ASM) method. a) seed points are first positioned within the cord and b) the boundary of the cord is identified to obtain cross-sectional area (CSA) measurements of the LSE c) the grey matter (GM) boundary and CSA is also obtained using semi-automated and manual editing techniques.

Image analysis was performed using JIM 6.0 (Xinapse systems, http://www.xinapse.com). A 15 mm section of the cord (i.e. 3 slices) was extracted and segmented based on previously described methodologies in the cervical spine [11], [16] as follows: Using the active surface model (ASM) segmentation method, seed points were manually positioned in the centre of the cord on all axial slices between T11-L1 and CSA measurements were obtained (see Figure 2a and 2b). In each subject, the slice with the largest CSA between T11-L1 was identified and the two adjacent slices were subsequently included in further analysis. GM segmentation was done based on a previously reported method [16], predominantly with the use of the fuzzy connector segmentation [25] available in Jim 6.0, but with manual editing whenever necessary to obtain the final GM contour (see Figure 2c and Figure S3). The CSA of WM (LSE-WM-CSA) was recorded as the difference between LSE-CSA and LSE-GM-CSA.

### Reproducibility assessment

Five out of ten study participants had three repeated scans, each performed on different occasions (at least one week apart), in order to test for ‘scan-rescan’ reproducibility; reproducibility was assessed by one experienced rater analysing all the data. In order to demonstrate intra-observer reproducibility, the same rater re-analysed all the data from the 5 volunteers' first visit 3 times; the analysis was done on separate occasions with a minimum of 2 weeks between each analysis. Inter-observer reproducibility was assessed by employing a second and third rater to analyse the data from the 5 volunteers' first visit.

Additional measures such us the Dice similarity coefficient (DSC) [26] and the modified Housdorff distance (MHD) [27] were also obtained. The DSC is a measurement of spatial overlap between two sets and represents the size of the union of two sets divided by the average size of the two sets; a value of 0 indicates no overlap while a value of 1 indicates perfect agreement. The MHD between two sets represents a measure of the distance between points in one set to the corresponding nearest points in the other set; lower MHD units (MHDu) indicate better registration.

### Statistics

Statistical analysis was performed using SPSS 11.0 (SPSS, Chicago, Ill., USA). For the assessment of intra- and inter-observer reproducibility as well as scan-rescan reproducibility, the coefficient of variation (COV) was calculated using the mean and standard deviation from the repeated measures and the equation COV =  [SD/mean] ×100%. In order to estimate the measurement error relative to the biological variability between subjects, the intra-class correlation coefficient (ICC) was calculated.

### Results

**Figure 3:**
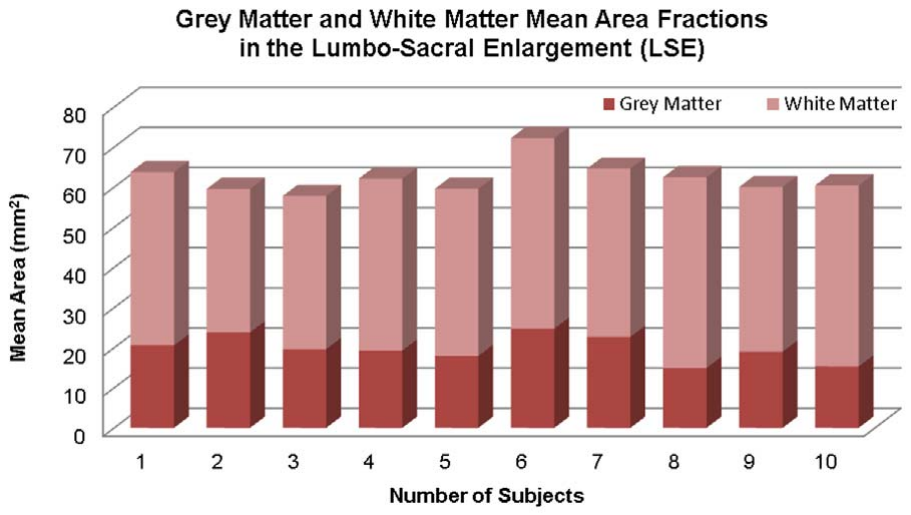
Stacked plot diagram showing grey matter (GM) and white matter (WM) mean area fractions measured in a 15 mm section through the lumbosacral enlargement (LSE) in 10 healthy subjects.

All the acquired images were included in the analysis and no data had to be discarded because of motion or other artefacts. The segmentation of the LSE-CSA was successfully performed using the ASM method without the need for manual intervention. However, the segmentation of the LSE-GM-CSA required manual editing in all cases. Mean (±SD) LSE-CSA of the 15 mm section studied (i.e. 3 slices) across 10 healthy subjects was 62.3 (±4.1) mm^2^ and mean (±SD) LSE-GM-CSA was 19.8 (±3.3) mm^2^. Figure 3 shows a stacked plot diagram of the mean GM and WM area fractions measured within the LSE in the 10 healthy subjects that took part in the study.

The mean scan-rescan, intra- and inter-observer % COV for measuring the LSE-CSA were 2%, 2% and 2.5%, respectively. The mean scan-rescan, intra- and inter-observer % coefficient of variation for measuring the LSE-GM-CSA were 7.8%, 8% and 8.6%, respectively. The ICC for LSE-CSA measurements was 0.76 and for LSE-GM-CSA measurements was 0.87.

**Figure 4:**
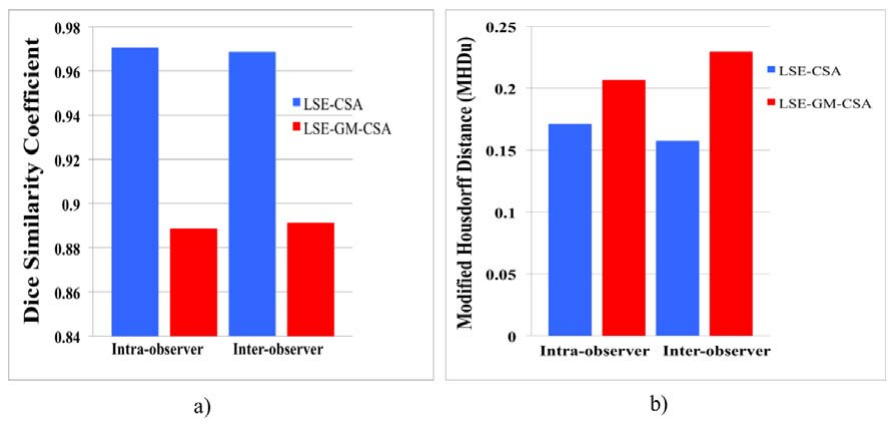
Image segmentation assessment. a) mean Dice similarity coefficient (DSC) of the lumbosacral enlargement (LSE) cross-sectional area (LSE-CSA) and the LSE grey matter cross-sectional area (LSE-GM-CSA) in 5 healthy subjects obtained from a single rater (intra-observer) and 3 raters (inter-observer) b) mean modified Housdorff distance (MHD) of the LSE-CSA and the LSE-GM-CSA.

Mean (±SD) DSC of the LSE-CSA (0.97±0.01) and the LSE-GM-CSA (0.89±0.01) for a single rater (intra-observer) in 5 healthy subjects and the mean DSC of the LSE-CSA (0.97±0.01) and the LSE-GM-CSA (0.88±0.01) for 3 raters (inter-observer) are shown in Figure 4a. Figure 4b shows the intra-observer MHD of the LSE-CSA (0.17±0.07) and the LSE-GM-CSA (0.21±0.07) and the inter-observer MHD of the LSE-CSA (0.16±0.02) and the LSE-GM-CSA (0.23±0.04). More details regarding the measurements of each observer and the corresponding similarity measurements can be found in Table S1, Table S2, Table S3, and Table S4.

## Discussion

In this study we have successfully presented a clinically feasible MRI protocol for obtaining tissue-specific (i.e. GM and WM) CSA measurements in the lumbar SC in healthy subjects using commercially available hardware and software, which has the potential for immediate clinical utility. The motivation for pursuing this study was found in the fact that cervical SC atrophy observed by measuring changes in CSA cross-sectionally and over time has provided valuable insights into disease state and evolution in MS and other important neurological conditions [9]–[14]. To date there are no clinical studies reporting such changes at lumbar level and there is little doubt that the development of imaging methods to study the lower SC will provide invaluable information to better understand the pathophysiology of commonly reported symptoms relevant to this level of the SC such as bladder and sexual dysfunction for example, which is a major problem not only in MS [28], [29] but in conditions like spinal cord injury (SCI) [30]–[36] and multiple system atrophy (MSA)[37], [38]. In MS, bladder symptoms are often attributed to spinal cord lesions, although in some cases no obvious lesions are present to account for bladder problems, raising the possibility that axonal degeneration may also be occurring in pathways that affect the bladder [28]. It is also evident that bladder problems in SCI may exist even in patients with relatively preserved neurological function [30] and medical imaging has offered very little so far in our understanding of these symptoms.

A number of hurdles unique to lumbar SC imaging had to be overcome to achieve these results. Firstly, the anatomical curvature and variation in this segment of the SC meant that a reproducible localisation strategy had to be implemented that was dependant on intrinsic SC markers. The LSE had been shown to be always located between the T11-L1 vertebral bodies [20] and as such, coverage of this section was deemed to be a pre-requisite in the final imaging protocol. The localisation technique further relied on identifying the widest section of the LSE, which is its mid-point, and in doing so reliably captured the lumbar segments of the SC. A minor drawback of this method, albeit unavoidable, was the requirement of using a sufficiently high number of slices to ensure coverage of the T11-L1 section with a consequent increase in the total scan time.

The optimisation of the 3D-FFE sequence parameters to achieve high image contrast involved the use of a low flip angle (α = 10°, for reduced T1 weighting) and short TR and TE (22 ms and 4.4 ms, respectively) for the acquisition of mixed proton density and T2* weighted images that have been shown to offer optimum contrast between the tissue types in previous studies of the cervical SC [16]. The voxel dimensions of 0.5 mm×0.5 mm in plane with a slice thickness of 5 mm was chosen to ensure reduced in-plane partial volume effects whereas the slice thickness was appropriate due to less variation in the longitudinal SC axis. In addition, SNR limitations due to the coil design and scan time restrictions meant it was not possible to consider acquisitions with reduced slice thickness. With dedicated coils it may be possible to increase SNR and improve through-plane resolution by reducing slice thickness. This would reduce the partial volume effect, which can affect the sharpness of the GM/WM boundary. Of course, SNR gain from dedicated coils may also be utilised in order to reduce the acquisition time and to make this protocol more acceptable in the clinical setting. In this particular protocol, a high number of averages was acquired (NEX = 8) in order to boost SNR. We investigated the option of magnitude averaging by acquiring 8 separate images to register and average in image-space, but the low SNR of each acquisition impaired the registration process, therefore k-space averaging was deemed appropriate.

Subject immobilization was absolutely essential to avoid movement artefacts and this was achieved by applying velcro straps across the torso and through the use of a standard MRI compatible cervical collar. Optimal positioning was achieved with the subjects lying flat with their knees bent at a 120 degree angle in the hip flexed position for improved contact of the lower back with the surface coil.

The reproducibility results demonstrated a mean scan-rescan, intra- and inter-observer % coefficient of variation for measuring the LSE-CSA of 2%, 2% and 2.5%, respectively and for measuring the LSE-GM-CSA; 7.8%, 8% and 8.6%, respectively. While there are no identical studies in the literature with which to directly compare these figures, these results are encouraging nevertheless as they are comparable to those obtained in the cervical SC, opening up the possibility to implement the method in the clinical setting using commercially available hardware. Horsfield et al. [11] demonstrated intra- and inter-observer COVs of 0.59% and 1.36%, respectively in cervical SC CSA measurements while Yiannakas et al. [16] have shown cervical SC CSA intra- and inter-observer COVs of 0.5% and 0.5%, respectively. In the original study by Losseff et al. [10], the mean scan-rescan COV was 0.8% in the cervical SC. The results of the present pilot study are promising and are in keeping with the results seen in the upper cervical cord CSA, although the small differences observed may be due to several factors, ranging from coil design differences and image resolution differences to the diameter of the cord itself, which has been shown from this study to be smaller in the lumbar SC than in the cervical SC, measured using similar methodologies [16].

With respect to GM COVs, the current work yielded values of 7.8%, 8% and 8.6%, respectively, which are different to previously reported values of 6.5%, 5.4% and 12.7% in the upper cervical SC [16]. The differences in COVs may be attributable to one, or a combination of factors such as partial volume effects which have a greater influence in GM estimation due to the tissue's smaller CSA; or the subjective GM segmentation that relied upon operator judgement rather than an automated process. At present there is no agreement on an established method for reliable segmentation of SC GM. The fuzzy connector method is a good starting point but manual editing was required in all cases. Although automated GM segmentation software could assist in improving the consistency of analysis, there would still be errors due to partial volume effects, which cannot exclude the need for manual outlining and correction of the masks.

In summary, this study has presented a new MRI acquisition and analysis protocol that uses the LSE as an intrinsic imaging biomarker, opening up the possibility of assessing neurological conditions that may differentially affect GM and WM in the lower SC. The protocol was developed on a clinical 3T system and is ready for immediate translation to further *in vivo* human studies. Further development will be focused on multi-modal quantitative MRI analysis and also on developing lumbar SC templates for registration purposes in view of possible group analysis.

## Supporting Information

Appendix S1 **Acquisition parameter optimisation.**
(DOCX)

Dataset S1 **Details of all measurements obtained in the reproducibility study.**
(XLSX)

Figure S1 **Imaging protocol optimisation and contrast-to-noise ratio (CNR) measurements.** a) plot demonstrating the effect of varying the echo time (TE) on grey matter (GM)/white matter (WM), by keeping the repetition time (TR) and flip angle constant, and the corresponding images at b) TE = 4.4 ms, CNR = 5.8 c) TE = 15 ms, CNR = 4.3 d) TE = 26 ms, CNR = 3.1 e) TE = 37 ms, CNR = 0.5.
(TIFF)

Figure S2 **Contrast-to-noise (CNR) calculation method.** a) example of region of interest (ROI) placement within white matter (WM) and grey matter (GM) on the original image b) interpolated image for better visualisation.
(TIFF)

Figure S3 **Image segmentation example of the lumbosacral enlargement grey matter cross-sectional area (LSE-GM-CSA) in two healthy subjects (a-b).** Top figures show the magnified original image, middle figures show the unedited LSE-GM-CSA contours (in white) and bottom figures show how these have been edited manually (final contours are shown in red).
(TIFF)

Table S1 **Inter-observer lumbosacral enlargement cross-sectional area (LSE-CSA) measurements (mm^2^).**
(DOCX)

Table S2 **Inter-observer lumbosacral enlargement grey matter cross-sectional area (LSE-GM-CSA) measurements (mm^2^).**
(DOCX)

Table S3 **Mean inter-observer similarity measurements of the lumbosacral enlargement cross-sectional area (LSE-CSA).**
(DOCX)

Table S4 **Mean inter-observer similarity measurements of the lumbosacral enlargement grey matter cross-sectional area (LSE-GM-CSA).**
(DOCX)
