# Supplementary material for: The Use of the Lumbosacral Enlargement as an Intrinsic Imaging Biomarker: Feasibility of Grey Matter and White Matter Cross-Sectional Area Measurements Using MRI at 3T
Source: PLoS One. 2014 Aug 29;9(8):e105544. doi: 10.1371/journal.pone.0105544 (PMC4149374; doi:10.1371/journal.pone.0105544)
Supplement: Appendix S1 — Acquisition parameter optimisation. (DOCX) [file pone.0105544.s001.docx]

Appendix S1. Acquisition parameter optimisation

For imaging the lumbar spinal cord (SC), a fat-suppressed 3D slab-selective fast field echo (FFE) product sequence was used. The optimisation of the 3D-FFE imaging protocol that was used for cross-sectional area (CSA) measurements involved repeated acquisitions of a single volunteer, and variation of the MR parameters each time to determine the most appropriate grey matter (GM)/white matter (WM) contrast. Considering previous reports of high GM and WM contrast imaging protocols in the cervical spine using PD/T2* weighted acquisitions [16, 24], optimisation of the 3D-FFE was carried out by employing a fixed low flip angle (α = 10°, to minimise T1 weighting) and the optimum image contrast was determined within the range of TR = 50 ms and the minimum achievable TE = 4.4 ms. Contrast-to-noise ratio (CNR) was measured from the images by placing 1.5 mm^2^ regions of interest (ROI) within GM and WM and using the equation:

$$CNR=\frac{\left(SI1-SI2\right)}{\sqrt{SD1^{2}+SD2^{2}}}$$

where, *SI*1 is the mean signal intensity in the ROI placed within GM and *SI*2 within the WM; *SD*1 and *SD*2 are the standard deviations in each tissue-type, respectively. CNR for each individual acquisition was calculated as the mean value obtained from three consecutive slices at the T11-L1 level. Figure S1 shows an example of CNR measurements and the effect of changing the TE while keeping TR and flip angle fixed. Figure S2 shows an example of ROI placement within GM and WM for CNR calculations.

Similar experiments were done by keeping the TE fixed at ‘minimum’ (TE = 4.4 ms) and varying the TR within the range of TR = 50 ms and the minimum achievable TR = 23 ms. CNR values within this range did not vary considerably (CNR = 5.1 - 5.8). For this reason, the shortest TR (TR = 23 ms) was used in the final imaging protocol because it offered reduced total acquisition time due to the lower TR.
