# Supplementary figures and images for: The Use of the Lumbosacral Enlargement as an Intrinsic Imaging Biomarker: Feasibility of Grey Matter and White Matter Cross-Sectional Area Measurements Using MRI at 3T
Source: PLoS One. 2014 Aug 29;9(8):e105544. doi: 10.1371/journal.pone.0105544 (PMC4149374; doi:10.1371/journal.pone.0105544)

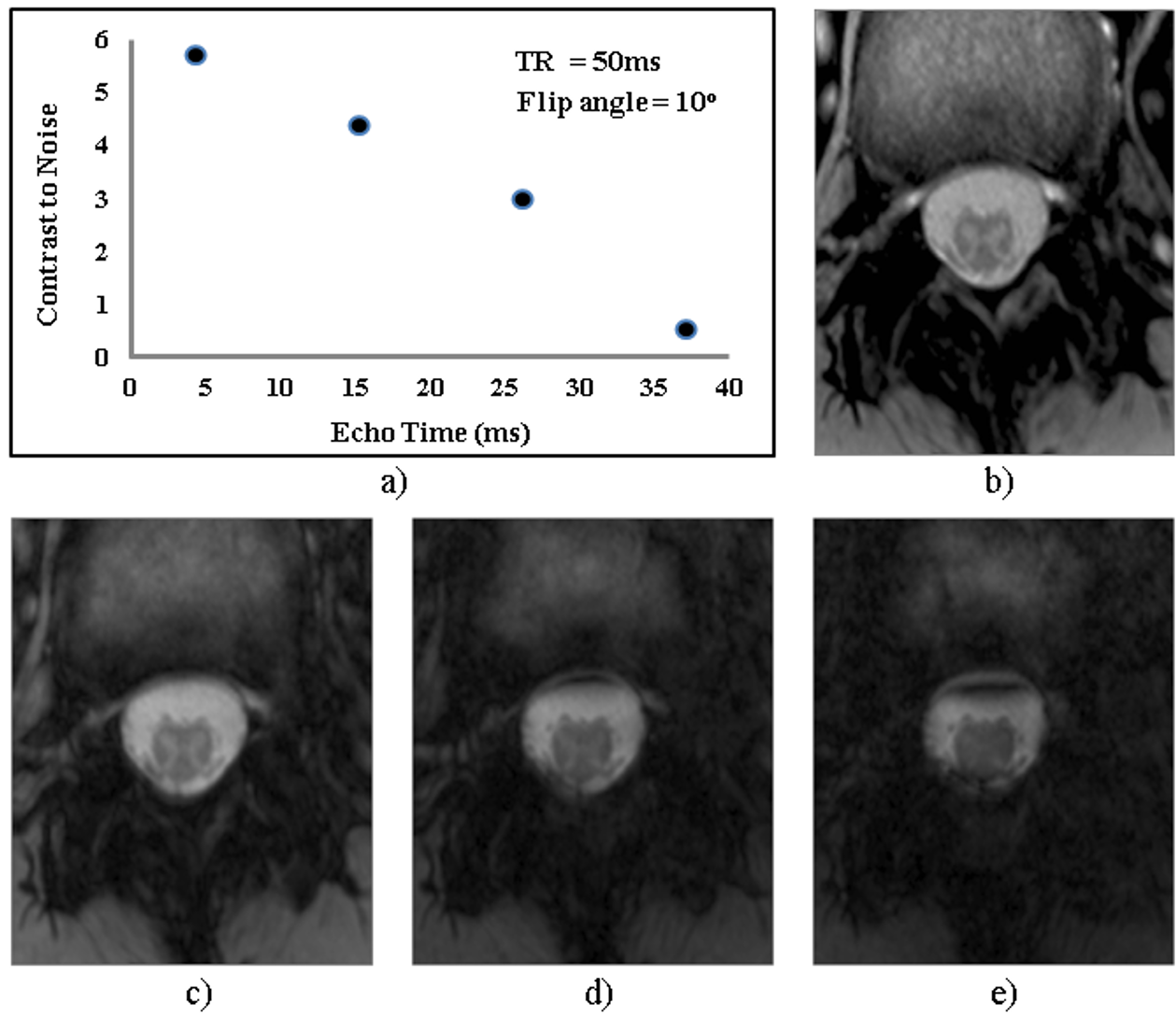

Supplement: Figure S1 — Imaging protocol optimisation and contrast-to-noise ratio (CNR) measurements. a) plot demonstrating the effect of varying the echo time (TE) on grey matter (GM)/white matter (WM), by keeping the repetition time (TR) and flip angle constant, and the corresponding images at b) TE = 4.4 ms, CNR = 5.8 c) TE = 15 ms, CNR = 4.3 d) TE = 26 ms, CNR = 3.1 e) TE = 37 ms, CNR = 0.5. (TIFF) [file pone.0105544.s003.tiff]

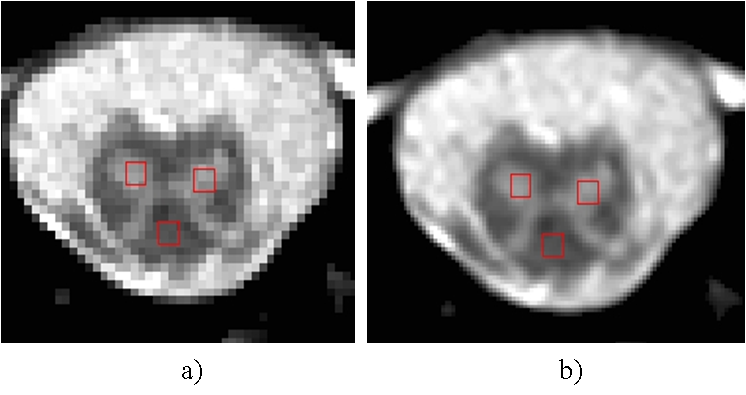

Supplement: Figure S2 — Contrast-to-noise (CNR) calculation method. a) example of region of interest (ROI) placement within white matter (WM) and grey matter (GM) on the original image b) interpolated image for better visualisation. (TIFF) [file pone.0105544.s004.tiff]

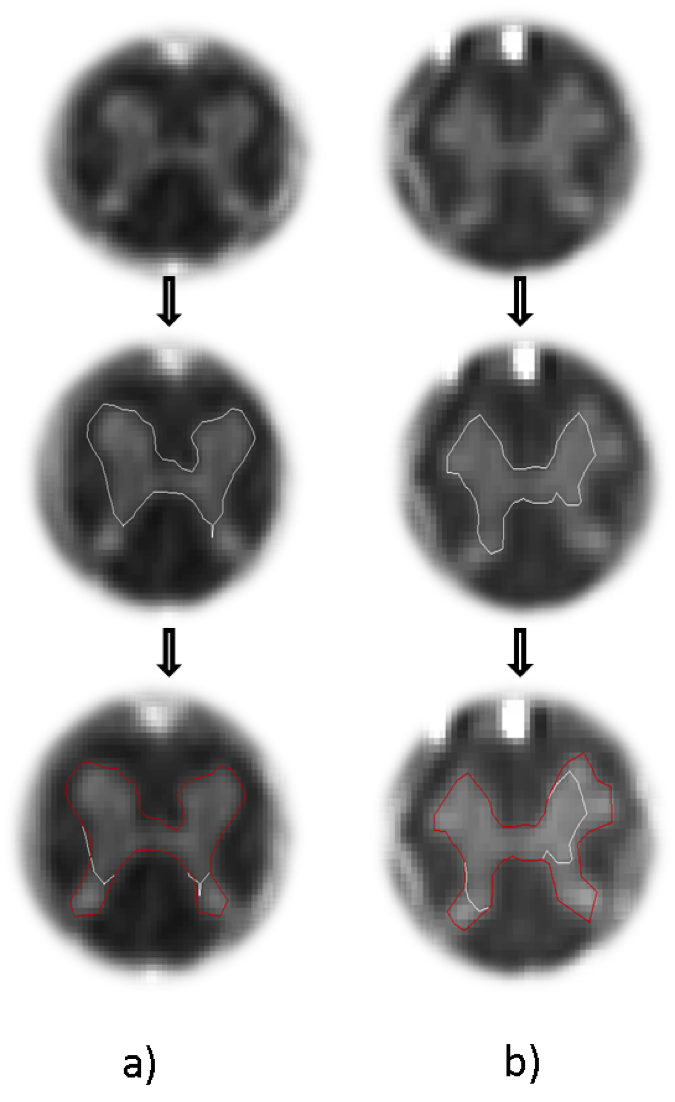

Supplement: Figure S3 — Image segmentation example of the lumbosacral enlargement grey matter cross-sectional area (LSE-GM-CSA) in two healthy subjects (a-b). Top figures show the magnified original image, middle figures show the unedited LSE-GM-CSA contours (in white) and bottom figures show how these have been edited manually (final contours are shown in red). (TIFF) [file pone.0105544.s005.tiff]
