# Supplementary material for: The Use of the Lumbosacral Enlargement as an Intrinsic Imaging Biomarker: Feasibility of Grey Matter and White Matter Cross-Sectional Area Measurements Using MRI at 3T
Source: PLoS One. 2014 Aug 29;9(8):e105544. doi: 10.1371/journal.pone.0105544 (PMC4149374; doi:10.1371/journal.pone.0105544)
Supplement: Table S1 — Inter-observer lumbosacral enlargement cross-sectional area (LSE-CSA) measurements (mm2). (DOCX) [file pone.0105544.s006.docx]

| Table S.1. Inter-observer lumbosacral enlargement cross-sectional area (LSE-CSA) measurements (mm^2^) | | | | | | |
| --- | --- | --- | --- | --- | --- | --- |
| Subject | Slice | Rater 1 | Rater 2 | Rater 3 | Variance | Mean |
| 1 | 14 | 66.54 | 66.41 | 65.28 | 0.48 | 66.08 |
| 1 | 15 | 61.92 | 61.47 | 58.46 | 3.54 | 60.62 |
| 1 | 13 | 62.82 | 64.92 | 61.89 | 2.41 | 63.21 |
| 2 | 14 | 59.81 | 64.00 | 63.31 | 5.05 | 62.37 |
| 2 | 15 | 59.20 | 63.17 | 61.03 | 3.95 | 61.13 |
| 2 | 13 | 58.90 | 62.47 | 59.97 | 3.36 | 60.45 |
| 3 | 12 | 58.53 | 56.83 | 56.01 | 1.65 | 57.12 |
| 3 | 11 | 56.99 | 56.24 | 53.65 | 3.07 | 55.63 |
| 3 | 13 | 57.85 | 56.83 | 55.59 | 1.28 | 56.76 |
| 4 | 10 | 63.03 | 62.43 | 60.28 | 2.09 | 61.91 |
| 4 | 11 | 61.46 | 61.67 | 59.60 | 1.30 | 60.91 |
| 4 | 9 | 61.90 | 61.97 | 60.17 | 1.04 | 61.35 |
| 5 | 12 | 60.12 | 60.44 | 62.95 | 2.40 | 61.17 |
| 5 | 13 | 59.73 | 60.25 | 61.94 | 1.34 | 60.64 |
| 5 | 11 | 58.93 | 58.13 | 60.53 | 1.49 | 59.20 |
