# Supplementary material for: The Use of the Lumbosacral Enlargement as an Intrinsic Imaging Biomarker: Feasibility of Grey Matter and White Matter Cross-Sectional Area Measurements Using MRI at 3T
Source: PLoS One. 2014 Aug 29;9(8):e105544. doi: 10.1371/journal.pone.0105544 (PMC4149374; doi:10.1371/journal.pone.0105544)
Supplement: Table S2 — Inter-observer lumbosacral enlargement grey matter cross-sectional area (LSE-GM-CSA) measurements (mm2). (DOCX) [file pone.0105544.s007.docx]

| Table S.2. Inter-observer lumbosacral enlargement grey matter cross-sectional area (LSE-GM-CSA) measurements (mm^2^) | | | | | | |
| --- | --- | --- | --- | --- | --- | --- |
| Subject | Slice | Rater 1 | Rater 2 | Rater 3 | Variance | Mean |
| 1 | 14 | 17.34 | 16.78 | 19.35 | 1.83 | 17.82 |
| 1 | 15 | 18.08 | 14.22 | 16.87 | 3.90 | 16.39 |
| 1 | 13 | 20.31 | 19.85 | 21.50 | 0.73 | 20.55 |
| 2 | 14 | 22.40 | 20.67 | 22.98 | 1.44 | 22.02 |
| 2 | 15 | 21.18 | 18.54 | 21.55 | 2.69 | 20.42 |
| 2 | 13 | 22.88 | 17.33 | 22.79 | 10.10 | 21.00 |
| 3 | 12 | 17.18 | 17.46 | 19.99 | 2.40 | 18.21 |
| 3 | 11 | 18.50 | 17.97 | 18.99 | 0.26 | 18.49 |
| 3 | 13 | 17.00 | 14.65 | 17.88 | 2.79 | 16.51 |
| 4 | 10 | 17.77 | 17.37 | 18.97 | 0.69 | 18.04 |
| 4 | 11 | 17.09 | 15.30 | 17.40 | 1.29 | 16.60 |
| 4 | 9 | 17.90 | 15.71 | 18.51 | 2.17 | 17.37 |
| 5 | 12 | 15.40 | 16.60 | 18.79 | 2.95 | 16.93 |
| 5 | 13 | 15.45 | 16.33 | 18.66 | 2.75 | 16.81 |
| 5 | 11 | 17.60 | 16.69 | 19.01 | 1.37 | 17.77 |
