# Supplementary material for: The Use of the Lumbosacral Enlargement as an Intrinsic Imaging Biomarker: Feasibility of Grey Matter and White Matter Cross-Sectional Area Measurements Using MRI at 3T
Source: PLoS One. 2014 Aug 29;9(8):e105544. doi: 10.1371/journal.pone.0105544 (PMC4149374; doi:10.1371/journal.pone.0105544)
Supplement: Table S3 — Mean inter-observer similarity measurements of the lumbosacral enlargement cross-sectional area (LSE-CSA). (DOCX) [file pone.0105544.s008.docx]

| Table S.3. Mean inter-observer similarity measurements of the lumbosacral enlargement cross-sectional area (LSE-CSA) | | | | | | |
| --- | --- | --- | --- | --- | --- | --- |
|  | LSE-CSA (mm^2^) | | |  | Measurement | |
| Subject | Rater 1 | Rater 2 | Rater 3 |  | DSC | MHD |
| 1 | 63.76 | 64.27 | 61.88 |  | 0.97 | 0.18 |
| 2 | 59.30 | 63.21 | 61.44 |  | 0.96 | 0.16 |
| 3 | 57.79 | 56.63 | 55.08 |  | 0.97 | 0.17 |
| 4 | 62.13 | 62.02 | 60.02 |  | 0.98 | 0.12 |
| 5 | 59.59 | 59.61 | 61.81 |  | 0.96 | 0.15 |

DSC: Dice similarity coefficient

MHD: Modified Housdorff distance.
