# Supplementary material for: The Use of the Lumbosacral Enlargement as an Intrinsic Imaging Biomarker: Feasibility of Grey Matter and White Matter Cross-Sectional Area Measurements Using MRI at 3T
Source: PLoS One. 2014 Aug 29;9(8):e105544. doi: 10.1371/journal.pone.0105544 (PMC4149374; doi:10.1371/journal.pone.0105544)
Supplement: Table S4 — Mean inter-observer similarity measurements of the lumbosacral enlargement grey matter cross-sectional area (LSE-GM-CSA). (DOCX) [file pone.0105544.s009.docx]

| Table S.4. Mean inter-observer similarity measurements of the lumbosacral enlargement grey matter cross-sectional area (LSE-GM-CSA) | | | | | | |
| --- | --- | --- | --- | --- | --- | --- |
|  | LSE-GM-CSA (mm^2^) | | |  | Measurement | |
| Subject | Rater 1 | Rater 2 | Rater 3 |  | DSC | MHD |
| 1 | 18.58 | 16.95 | 19.24 |  | 0.88 | 0.29 |
| 2 | 22.15 | 18.85 | 22.44 |  | 0.9 | 0.22 |
| 3 | 17.56 | 16.69 | 18.95 |  | 0.9 | 0.19 |
| 4 | 17.59 | 16.13 | 18.29 |  | 0.9 | 0.24 |
| 5 | 16.15 | 16.54 | 18.82 |  | 0.8 | 0.21 |

DSC: Dice similarity coefficient

MHD: Modified Housdorff distance.
